# Increasing efficiency in vaccine Production: A primer for change

**DOI:** 10.1016/j.jvacx.2021.100104

**Published:** 2021-06-16

**Authors:** Ole Kristian Aars, Michael Clark, Nina Schwalbe

**Affiliations:** a Spark Street Advisors, New York, NY, United States; b Mailman School of Public Health, Columbia University, New York, NY, United States; c University of Oslo, Oslo, Norway; d University of the Witwatersrand, Johannesburg-Braamfontein, Gauteng, ZA, South Africa

**Keywords:** Research and development, Global health, Manufacturing, Second-generation vaccines, Vaccine access

## Abstract

The COVID-19 pandemic has highlighted the importance of vaccines as public health and pandemic preparedness tools and amplified the importance of issues ranging from equitable distribution to reliable supply of quality, affordable vaccines. These issues however are not new. Delays in time from the first dose in a high-income country to introduction at scale in a low-income country can take years. These delays are driven by several challenges, some of which are unique to the vaccine development ecosystem. The patenting and overall intellectual property (IP) protection are complex, regulatory oversight is rigorous, manufacturing processes require technical support or know-how transfer from the innovator, and market dynamics create obstacles to delivering at scale. However, there are opportunities to accelerate the introduction of vaccines at scale in low and middle-income countries. To identify those opportunities, this paper provides an overview of the vaccine research and development process and where reform of the current system could increase access.

## 1 Definitions and background information

Vaccines are biological products^2^ produced through the manipulation of genetic material and living organisms such as cultures of animal cells, yeast, bacteria, insect cells, or host organisms (fertilized eggs). The fact that they are biologicals makes many aspects of development, production, regulatory constraints and patent structure distinct from small molecule medicines [2], [3]. In contrast to generic medicines that are chemically identical, most vaccines produced with the same active components are in effect a “new” entity. Thus, even so-called second-generation vaccines require human studies to demonstrate safety and efficacy, and development and manufacturing steps undertaken by the innovator must be replicated by the new entrant to prove equivalence or non-inferiority to the originator.

## 2 The vaccine development process

The main steps required to bring a vaccine to market are research and development, manufacturing scale-up, and regulatory approval.

**Research and Development** (R&D) begins with preclinical development in the laboratory and is increasingly often the subject of patent filing. If a preclinical candidate is deemed to have the potential for use against a particular disease after testing in animal models, clinical trials follow to test dosing and safety in humans (phase I), before immunogenicity and efficacy are assessed (phase II) and efficacy and safety further established with increasing human sample sizes (phase III). If the product is deemed both efficacious and safe, the company applies for licensure and registration. This is followed by ongoing pharmacovigilance (phase IV), which is the process of monitoring adverse events post-marketing approval [3].

**Manufacturing scale-up:** As a product enters phase I trials, developers should begin to identify the necessary steps to produce at scale to meet future demand, as quantities for phase I arelimited to only what was required for testing in a laboratory. Refinements to the production process are made as part of both clinical and commercial scale-up. The biological processes are often difficult to replicate at scale and quality assurance and quality control (QA/QC) are therefore an integral part of scale-up. After licensing, production improvements continue.

**Regulatory:** Regulatory authorities set guidelines for how trials are conducted and give developers authorization to conduct trials in humans (in the “clinic”) based on successful results in the lab (“preclinic”). After having undertaken preclinical and clinical testing, data on safety and efficacy is submitted to the relevant regulatory body for licensing. After a market authorization is granted, regulators oversee lot release and manufacturing site inspections to ensure that products and facilities continue holding the same standards. Post licensing product improvement is also subject to regulatory scrutiny and post-marketing requirements.

**Market development:** Throughout the R&D and production processes, a developer will file for patents as it acquires a new understanding of the behaviour of the product under development. If approved by the relevant patent office, the patent holder is granted a time-limited monopoly on sales, manufacturing and other activities, including follow on innovation. Since filings will often take place early in the development process, prior to market authorization, the effective patent time (time that the patent holder has exclusivity on sales and other activities) will be shorter than the actual patent time [4]. Considerations on where to market the product start early in the development process, taking into account market potential. The decision also depends on likely procurers of the product (i.e., national agencies, multilateral entities and or private market), production capacity, pricing structures, and logistics. These same factors inform whether a new manufacturer may want to engage in the same disease or area.

## 3 Key actors in vaccine R&D

Several different actors are engaged in vaccine R&D, although roles have shifted over time with the consolidation of multinational corporations (MNCs), a proliferation of biotech companies and the entrance of developing country manufacturers to supplying global markets.

### 3.1 Research and development (R&D)

Government entities play a key role in advancing product development, but primarily with a domestic mandate and often focused on early-stage development. For example, the European Commission and the Biomedical Advanced Research and Development Authority (BARDA) and the National Institutes of Health in the US are also major funders of vaccine research, as are several other governments (i.e. Germany, Japan, France) – either through dedicated agencies or grants made to privately owned entities.

There are two major groupings of vaccine manufacturers. MNCs are for the most part organized under the “International Federation of Pharmaceutical Manufacturers & Associations” (IFPMA), a trade group that represents companies involved in both therapeutic drugs and vaccines. Vaccine manufacturers from developing countries for the most part belong to the “Developing Countries Vaccine Manufacturers Network” (DCVMN). MNCs previously controlled most parts of the vaccine research and development processes, but their dominance is diminishing.

There is a growing presence of academic and private biotechnology players in the research field driving a large part of innovation. This means that MNCs are increasingly focused on development, registration, and manufacturing. These activities require large amounts of capital and play to their strengths. As a result, MNCs’ development pipelines are more and more being replenished from external sources through in-licensing and occasionally through mergers and acquisitions [5]. Another trend is the increasing success of DCVMNs to rapidly develop complex second-generation vaccines without formal technology transfer (i.e. pneumococcal conjugate vaccines (PCV), hexavalent, and human papillomavirus vaccine (HPV)). Finally, there have also been significant advances in vaccine science such as structural biology, protein engineering, immunology, and manufacturing platforms, among others [6]. These trends, together with the intensity of activity and discussion around COVID-19 vaccine research and access, may lead to considerably more manufacturers in the coming decades.

Biotech firms, academia and public research entities conduct much of the early research but may not have the financial backing, incentives, or competence to generate sufficient data and take products through clinical development and licensure. Where the growth in the number of new vaccine development programs amongst MNCs has stagnated over the last 15 years, amongst small biotechs it has more than doubled, and emerging-market players saw a 13-fold increase [7]. While on one hand this suggests increasing competition for MNCs, most of the programs have focused on second-generation vaccines, changing the vaccine manufacturing landscape. Looking at the landscape of novel COVID vaccine candidates, we also find many that have been taken forward by smaller biotechs [8]. As such, the pandemic might be a further catalyst for changing the landscape.

Despite more limited financing and in-house technical capabilities to develop new products, DCVMN manufactures are also increasingly capable of R&D, in part because of the ability to bring in organizations with specialized capabilities for different parts of the development process. An early example was the meningococcal A conjugate vaccine (MenAfriVac). With investment from the Bill & Melinda Gates Foundation (BMGF) and technical assistance from PATH, the Serum Institute of India developed the first-ever vaccine for Africa and is now the sole global manufacturer [9].

Another important contributor to the development of vaccines for low-income markets was the “product development partnerships” (PDPs), set up nearly 20 years ago by the BMGF and Rockefeller Foundation, among others, to target neglected diseases or diseases of poverty (i.e., malaria, tuberculosis, and HIV/AIDS). The scientific challenges of developing vaccines for these diseases are formidable, which is reflected in the fact that few new products have been brought to market over the last two decades. For example, it took 30 years for GSK to develop its Mosquirix vaccine due to the complex biological nature of the malaria parasite [10]. Despite the lack of progress in licensed products, however, these partnerships are considered highly successful in terms of galvanizing their respective fields of work and bringing attention to the need to develop products for neglected diseases [11].

Beyond disease-specific R&D, some organizations are developing “enabling sciences”. This refers to challenges that are not unique to one entity but may help accelerate development timelines or ease comparison across similar biologicals entities [12]. For example, investments in diagnostics are critical for case detection in clinical trials during outbreaks [13], epidemiological surveillance capabilities are crucial for needs-forecasting, the establishment of harmonized biological standards and assays is important to compare products across geographies [14] and better animal models can improve validation of proof of concept [12] and thus speed development for products for a range of diseases. CEPI, the Coalition for Epidemic Preparedness Innovation, has been especially active in this area with investments in particular with regards to the development of harmonized biological standards, assays and animal models.

### 3.2 Manufacturing

Manufacturing vaccines is very resource-intensive, whereby government-backed industries and larger MNCs – at least traditionally – have been the main entities with the capabilities required. Whereas previously manufacturing was an integrated part of a vaccine developer's portfolio, it is now increasingly being outsourced, including to so-called “contract manufacturing organizations” (CMOs) – a market that has been valued at $1.8bn in 2016 [15]. The ability of these organizations to help with quality, reliability and regulatory issues have been highlighted as some of the main drivers of the trend to outsource [16]. Outsourcing these functions can also lower facilities cost and financial risk in early project stages (e.g. pre-clinical and phase I/II) and then switched to in-house for phase III/ commercialization when the level of financial risk is lower and greater control over the product is required.

The largest manufacturer from a volume perspective (e.g. the number of doses) is Serum Institute of India, whose output almost exceeds that of its three closest competitors. In terms of value, however, MNCs’ (Merck, Pfizer, Sanofi and GlaxoSmithKline GSK) substantial footprint in high-income markets means that they rank considerably higher despite producing a lower volume. Several government-controlled organizations also have large manufacturing capabilities that cater to domestic and sometimes regional demand. Examples include the government-backed entities of China (Chengdu Institute of Biological Products Co), Russia (Chumakov Federal Scientific Center for Research), Indonesia (Bio Farma), and Brazil (Fiocruz and Instituto Butantan) [17]*.*

While all pharmaceuticals require batch testing, the risk of batch failure is higher for vaccines than for most other pharmaceuticals due to the intrinsic biological variability of most materials and interactions between them [2]. Batch release processes can also lengthen the time required for manufacturing as they often require in-vivo^3^ testing.

Likewise, the sensitive nature of biological materials requires the developer to demonstrate consistency, quality control and quality assurance for the entire process of manufacturing, including the critical stage of scale-up. Generally known as “Chemistry, Manufacturing and Controls” (CMC) [18] these processes can require investments exceeding USD 50 m and more than 80 person-years in terms of labour [3]. This fixed investment represents a significant barrier for second-generation vaccine developers. Further, all manufacturers also need to develop lot-testing procedures that include the analysis of potency, safety, and purity. These may include in-vitro and in-vivo tests that take weeks or months to conclude.

### 3.3 Regulatory engagement and approval

Regulators determine whether a vaccine is sufficiently safe and effective to enter and stay in the market. They also periodically inspect the manufacturing facilities for “Good Manufacturing Practices” (GMP) and QA/QC. Regulators are also now experiencing and expanding roles as requirements towards safety and efficacy have increased, as have technological innovations, such as platform technologies^4^, adjuvants, and injection devices, that also require the development and application of new regulatory pathways [19]. For example, some regulators may require additional clinical studies if the target population in their respective settings differs from those tested in trials[20]. Applications for market authorization are reviewed either by the national regulatory agencies (NRA) where the manufacturer operates, by the centralized procedure if in the European Union (EU), or by a Regulatory Authority of specific choice if the manufacturer so chooses. European countries have mutual recognition of assessments made by other member states under the auspices of the European Medicines Agency (EMA) [20]. Similarly, the African Vaccine Regulatory Forum (AVAREF) attempts to drive alignment through engagement between NRAs on the continent, but without regulations that allow for the mutual recognition process that the EMA has instated [21]. CEPI has also established a regulatory working group, aiming to drive progress in the area.

WHO also plays an important role through its prequalification process; a comprehensive assessment of whether a vaccine meets requirements for safety and efficacy in immunization programs, and the assessments of the functionality of the releasing national regulatory authority [23]. WHO prequalification is a requirement for purchase by United Nations agencies (e.g. PAHO, UNICEF). Prequalification is paid by the manufacturer through a fee system and is done on a product-by-product basis for both innovator and second-generation vaccines.

### 3.4 Vaccine market development

Before the 1990 s, the vaccine market was considered non-attractive by large pharmaceutical companies and smaller players did not have the technology or financial capacity to get involved. Over the past 25 years, the global vaccines market has grown in terms of value and volume in part due to the launch of several “blockbuster” vaccines such as hepatitis B, multivalent DTP, pneumococcal, HPV and zoster. Another driver behind this trend is the establishment of procurement and funding partnerships that enable access to vaccines for low and some middle-income countries.

Gavi, the Vaccine Alliance, the largest of these initiatives, now procures vaccines for nineteen diseases, up from six diseases when it was first established in 2001. Their success in increasing access to vaccines has to a large extent been enabled the Alliance’s ability to negotiate “tiered pricing” arrangements. Practically, manufacturers agree to sell large volumes of vaccines to eligible lower-income countries for a price significantly lower than for middle-income and higher-income countries[24] . For example, for Gavi-eligible countries in WHO’s Africa region, the price for HPV vaccines from GSK in 2018 was on average USD37m versus USD114m for non-Gavi countries [17]. While countries that have never been eligible for Gavi support do not have access to these lower tiers of pricing, countries that are transitioning from Gavi support are granted access to continued concessional pricing for a period of time to avoid sudden budgetary impacts from assuming responsibility for vaccine procurement [25]. This policy was adopted after some criticism that countries would face “price walls” as increases in the per capita income moved them out of eligibility for Gavi support. Gavi, together with UNICEF, the Gates Foundation, and others also prepares supply and procurement roadmaps [26] for each vaccine in the Gavi portfolio, which identifies actions required to sustain or increase affordability.

Market concentration in vaccine procurement is driven by logistical complexities (i.e., the need for cold chain), economies of scale (large shipping volumes), and requirements for stockpiles. This has resulted in countries primarily relying on three different pathways; i) self-procurement/regional procurement, ii) the Pan America Health Organization’s (PAHO) Vaccine Revolving Fund and iii) UNICEF’s Supply Division (UNICEF-SD). Low-income countries (and some lower-middle-income countries) are primarily supplied vaccines through UNICEF-SD and PAHO. UNICEF-SD and PAHO also serve as the primary procurement partners for Gavi.^5^ Most upper-middle and higher-income countries conduct self-procurement and financing through national agencies [17], resulting sometimes in important access gaps.

### 3.5 Normative guidance and related WHO processes

While decisions around vaccine adoption and schedule are ultimately sovereign, some players influence those decisions by providing guidance on immunization policy. In particular, the WHO through its Strategic Advisory Group of Experts on Immunization (SAGE) and Regional Immunization Technical Advisory Groups (RITAGs) are important in this regard. In addition to reviewing efficacy and effectiveness, recommendations from these groups can include considerations of costs, cost-effectiveness and appropriateness of presentation for the setting or conditions in which the vaccine will be deployed . Recommendations may be directed towards a specific manufacturer or applicable for a disease area [27], [29]. All vaccines are assessed regardless of whether they are an innovator or second-generation.

WHO also plays an important role through the development and publication of a vaccine R&D Blueprint. The Blueprint is aimed at improving coordination and accelerating R&D for diseases that are considered to pose the greatest public health risk due to their epidemic potential [30]. The Blueprint served as the basis for CEPI’s decision making around its current disease focus.

There are also specialized procedures in place for new or unlicensed products for use in emergencies. For example, the U.S. Food & Drug Administration (FDA) and WHO have established the Emergency Use Authorization (EUA) [31] and the Emergency Use Listing Procedure (EUL) [32] respectively, helping to accelerate regulatory assessment and use of urgently needed products during a pandemic, epidemic or outbreak.

## 4 Key barriers to “time to market” and opportunities to drive efficiencies

Vaccine development takes place in a complex ecosystem with many actors involved. The biological nature of the products and the associated regulatory requirements make it even more difficult to accelerate the process. The result is that vaccine R&D can be lengthy and costly, which can create challenges for accessibility and affordability[1]. While acceleration is of development can be related to adequacy of funding, here we focus on some of the more structural challenges that can either incentivize or create opportunities to speed development, from discovery to delivery.

### 4.1 Research & Development (R&D)

Both second generation as well as innovator vaccines [3] share barriers to accelerating development. By using indirect biomarkers, correlates of protection^6^ is an opportunity to avoid larger efficacy studies. However, few vaccines can rely on this method alone to guarantee efficacy [33]. Together with the fact that vaccines are mostly given to healthy recipients to avoid a potential disease, this results in large, complex and costly trials to produce sufficient statistical evidence to prove protection [3]. Some of these challenges can be met through innovative trial designs, as was seen in the Ebola crisis. In this case, the development timeline was truncated substantially due to a design that allowed for parallel implementation of phase I, II and III trials [34]. Although such advancements are often made during emergencies, they could be considered more broadly or in particular for diseases of poverty.

While regulatory oversight must be maintained, there are also emerging R&D opportunities in the form of new technologies and enabling sciences that have the potential to either leapfrog typical steps or cut development timelines and costs found in standard pathways. For example, the development of new vaccine antigens and biological standards and assays can help accelerate proof of concept [44]. Moreover, new adjuvant technologies can strengthen and/or orient the immune response, reducing the frequency of vaccination and facilitating dose sparing, helping reduce the cost of vaccine doses [35], [36]. As demonstrated in the fight against COVID-19, the use of viral vector technology has proven promising and 13 candidate vaccines are using this technology [37]. Potential advantages include strong immune response, “plug and play” development and easily replicable manufacturing [38].

With regards to the approach to vaccine development, mRNA vaccines have the potential to revolutionize the field [39]. DNA vaccines also present a novel approach, but the field has not advanced as far. The basic principle of these vaccines is to deliver DNA or mRNA that codes for selected antigens of the target pathogen; the recipient person's cells will synthesize the coded proteins and the immune system will recognize and act against them. There are three main benefits of such a system: i) research steps are considerably simplified and accelerated, ii) production methods are non-pathogen specific and therefore standardized, flexible, and potentially rapid and easy to scale up and iii) it may provide a more flexible way of adjusting the vaccine if the pathogen mutates or changes.

Among the 76 vaccines against SARS-CoV-2 in clinical development as of March 2, 2021, nine are mRNA-based and eleven are DNA-based [37]. These technologies, if confirmed successful mid-to-long-term, could represent a significant potential for new vaccines made faster at lower production costs. As such, the recent roll-out by BioNTech/Pfizer and Moderna of their mRNA vaccines against COVID-19 is a landmark. While DNA vaccines have not shown the same progress, there has been positive development (i.e. Inovio) [40]. Given the engagement of many public funders in this field, access agreements upfront in the R&D process, including around management of IP and know-how, transfer of technology and pricing considerations, will be particularly important to monitor. The IP landscape for these vaccines have already proven to be highly fragmented [41], and given the nascency of the technology, developers might take a restrictive approach to participating in technology transfer or granting IP access. The WHO initiative to establish a COVID-19 patent pool is such a promising development [42].

### 4.2 Manufacturing

Some of the challenges related to the manufacturing of vaccines apply to all vaccines and others are specific to a particular vaccine class [3]. The fine-tuning of each step in the production process takes years to develop and is often done in parallel with preclinical and clinical development. Associated scale-up involving experiment-based adjustments are therefore not available to or discoverable by new players in the field. Further, production facilities must be dedicated or reconfigured for other biological entities. Manufacturers also need to develop and implement lot-testing procedures that include the analysis of potency, stability and purity that can delay timelines. Together, this complex range of production steps is often referred to as “know-how”. This know-how is probably the most critical barrier to the speedy development of second-generation vaccines and may, at least partly, be addressed through technology transfer.

In a report by the WHO, recipients of technology transfer highlighted that insufficient R&D capacity and human resources to support and demonstrate successful technology transfer were among the main obstacles [43]. Some efforts have been initiated to tackle this, including the establishment of know-how and other intellectual property pools and technology transfer hubs to facilitate handover, or requiring that recipients of public funding (e.g. CEPI) support technology transfer efforts. Despite these efforts, there have been few agreements for technology transfer for second-generation vaccines [2]. The potential of technology transfer to reduce costs and time to market in low-income countries as well as increase volumes and overall access remains largely untapped and this could be a key area for future reform and one where a neutral organization could consider a role in brokering partnerships and advising on deal structures. With support from donor and philanthropic organizations like PATH, DCVMN related vaccine manufacturers are increasingly developing in-house technical skills and know-how which over time may help address this issue. This is an area that could benefit from further investment, as could facilitation of discussions and agreements (e.g. IPR, technology transfer) between targeted DCVMN manufacturers that do not have access to other regional markets and potential DCVMN players in those regions.

### 4.3 Regulatory

Regulatory agencies play a vital part in ensuring safe and effective vaccines, but also face difficult trade-offs; on one hand by asking for additional evidence, they keep harmful or ineffective drugs from entering the market; on the other, the time advisable to perform the due diligence may lead to potentially foregoing health benefits by delaying access. Vigilance in evaluating vaccines is well-founded, but not without opportunities for improvements. Contrary to most traditional medicines, some vaccines require that producers meet biosafety level (BSL) standards because they can include the manipulation of dangerous pathogens during the production process. This may require biological hazard and biosafety level certification, prevention of contamination, cell line management, sourcing of raw materials (for example, specific-pathogen-free eggs), freeze-drying capacity/fine-tuning, conjugation technology, access to non-standard adjuvants, etc.

It also requires an in-depth understanding of the development process, which contains additional layers of approval as compared to small molecules. Approval is granted usually by the NRA where the manufacturer operates, by a centralized procedure if in the EU, or by a Regulatory Authority of specific choice if the manufacturer so chooses. If targeting low and middle-income countries, a WHO Prequalification is required. This can take up to 12 months [3]. Batch release also requires NRA approval, and NRA must periodically inspect the manufacturing facilities for “Good Manufacturing Practices” (GMP), and QA/QC. Both of these require that the NRA have adequate competencies and resources for product assessment. To address these issues, there are several global efforts underway to harmonize regulatory processes across geographies from clinical trials through to registration and beyond. The African Vaccine Regulatory Forum (AVAREF) is an example of this for clinical trials and the WHO's prequalification system for lot release [22]. While this does not obviate critical steps such as biosafety requirements related to batch release, it should speed time to market. Such efforts are especially important for vaccines with potential for high public health impact.

### 4.4 Market development

In theory, intellectual property incentivizes companies to make substantial investments in R&D and manufacturing. However, it also deters new entrants – both for existing products that are off-patent and for those that build on previous innovations [45].

Patents and other intellectual property protections for vaccine manufacturers are numerous and diverse and address most of the specificities of vaccine “knowledge” and “know-how” (trade secrets). For example, a study done in 2012 by the World Intellectual Property Organization [46] found that 11,800 patent families had been filed between 1921 and 2011 for active constituents of prophylactic vaccines against infectious diseases. Among these, 516 patent families related to flu vaccines, 165 patent families related to pneumococcal conjugated vaccines, and 36 patent families related to typhoid conjugated vaccines. Patents include technologies required for manufacturing (cell cultures, nucleic acid sequences, viral vector technology, virus/cell bank technologies, egg-based virus replication, etc.), active ingredients (antigens, adjuvants), production technologies, and processes (e.g., filtration, purification, conjugation, freeze-drying, etc.). The safeguarding of know-how and patenting of smaller components of the vaccine development process can be important to vaccine manufacturers as it extends protection in time far beyond the initial patent for active ingredients. However, when such patenting does not reflect substantial technological advancements, it may lead to unreasonably long patent protection. Such an unjustified extension of the exclusivity period can impede price competition and follow-up innovation, ultimately limiting access to potentially life-saving products [45]. This is an area that could benefit, for example, from additional scrutiny of regulatory authorities granting of an extension of protections.

Since patents are, for the most part, not transferrable across jurisdictions, vaccine developers will often need to file their patents in multiple jurisdictions. Specific licenses are also required before exporting a vaccine; for example, the importing country may require the manufacturer to conduct country-specific clinical trials [3]. Such barriers add to the difficulties around logistics and production economics for vaccine manufacturers [47]. Some patents are also the subject of complex licensing cross-agreements and royalty payments between several intellectual property rights (IPR) holders as a result of negotiations to terminate disputes [44]. This may generate high financial stakes for several patent holders and make potential IPR negotiations further complex [3]. For example, Bio-Manguinhos had to pay 4–5% running royalties on the Hib vaccine to GSK [48].

Despite the growth in manufacturing in India and China in recent years [49], [50], the IPR landscape for vaccines – from discovery to delivery – creates substantial obstacles for new players [2]. Understanding the landscape requires costly expertise, resources, and a capacity to undertake difficult and lengthy navigations around IPR. This could be in part addressed by a dedicated clearinghouse that focuses on reducing IPR-related barriers for DCVMN manufactures and potentially facilitates access to patent licenses. Activities to consider include: the creation of a vaccine patents database on strategically selected areas; supporting targeted freedom to operate analyses and solution-based resource tools; and, potentially, support DCMVN manufacturers with skills like legal expertise and negotiation strategy.

### 4.5 Costs

As discussed, there is no such thing as a “generic vaccine.” This makes the economics and market dynamics of vaccines different compared to small molecule medicines. This means that even when patents expire, much of the large fixed costs cannot be avoided for new entrants, creating an added barrier to competition. The originator may, depending on the time to market, have recouped its investments during the monopoly period that the patent(s) have granted, allowing an opportunity to reduce the price over time to closer to marginal cost, if there is competition [2]. Since new entrants are forced to undergo many of the same upfront investments, the large initial fixed costs may make it difficult for them to compete on price unless tech-transfer partnerships are entered into. As a way to address this, the BMGF and governments have provided so-called up-front “push funding” and incentives for many DCVMN companies working on second-generation vaccines for low-income countries to offset fixed and development costs (e.g., pneumococcal, pentavalent, rotavirus). Also, some DCVMN business models, in particular those with partial government ownership, can allow for lower profit margins than a private or shareholder-based structure.

## 5 The way forward

In this paper, we have highlighted actions that can be taken at each stage from the process to accelerate access. From R&D, to manufacturing and regulatory, to the management of incentives like patents and public funding, there are ways to speed time to market and decrease costs. Examples highlighted include development of correlates of protection, innovative trial designs, enabling sciences such as adjuvant technologies, new technology approaches like mRNA vaccines, patent pools, conditions attached to public funding, materials sharing and technology transfer hubs and agreements, regulatory harmonization, and upfront push funding to offset fixed costs.

The COVID-19 outbreak and steps that have been taken to speed time to market could act as a catalyst for other vaccines, including new products and second-generation vaccines.

First, regulatory agencies are making use of specialized procedures for accelerating access. Just recently, the FDA made use of the EUA procedure to accelerate approval of COVID-19 vaccines[31]. With this has come the strong recognition of a need for harmonization of requirements by different regulatory agencies around the world, which will enable efficiencies to save both time and money. This is particularly important with regard to new technologies. For example, mRNA and DNA vaccines have the greatest potential of speeding the development processes [6] and the recent approval of mRNA vaccine against COVID-19, brings promise for fully establishing regulatory pathways for these innovations. While mRNA vaccines do pose some challenges for low-income settings, in particular their requirement for ultra-cold storages, there are ongoing efforts to try to address this issue, including the establishment of a COVID-19 mRNA vaccine technology transfer hub [51].

Second is the recognition that for vaccines to be deployed quickly and at scale, manufacturing capacity must be in place to allow for sufficient scale up when demand is high – as seen during the current pandemic. Given the substantial know-how required, access to facilities is not enough; low-income countries and regions must also have sufficient know-how about manufacturing processes [52]. This will require freedom to operate around patents and investment in technology transfer. To face the unprecedented need and opportunity for rapid and massive worldwide availability of COVID-19 vaccines, new business models have emerged with agreements between originator companies and manufacturing companies operating in different geographical and market environments. These precedents may pave the way towards a more flexible ecosystem involved in the continuum of vaccine production.

Third, with regards to IP arrangements, biopharmaceutical manufacturers and governments have made use of governmental compulsory licensing, patent oppositions, IP pools and voluntary IP licenses and technology transfer to advance access to new technologies. Although this has been limited in the field of vaccines, because of the issues discussed [53], improving transparency and creating more streamlined IP arrangements, could contribute to increased diversity of supplier which will also help alleviate supply constraints.

While still very much a “work in progress“ the advancements demonstrated through the R&D of COVID-19 vaccines, give promise that many of the issues outlined above can be successfully addressed with adequate financing and political will.

## Funding

The development of this paper was supported in part with funding from the Medicines Patent Pool.

## CRediT authorship contribution statement

NS conceptualized the manuscript. OA and NS wrote the manuscript. MC reviewed and edited the manuscript. NS and OA are joint first authors.

## Declaration of Competing Interest

The authors declare that they have no known competing financial interests or personal relationships that could have appeared to influence the work reported in this paper.

## Footnotes

2 “Biological” refers to either one or several components of the vaccine, or the method of production.

3 “In vivo” refers to inside a living organism, as opposed to “in vitro” which is outside a living organism

4 A vaccine candidate is platform-based if “an underlying, nearly identical mechanism, device, delivery vector, or cell line [is] employed for multiple target vaccines” [54].

5 In Latin America, PAHO serves as Gavi’s procurement partner.

6 A correlate of protection is a biological marker, such as an antibody level.
